# Antioxidants and Vasodilators for the Treatment of Noise-Induced Hearing Loss: Are They Really Effective?

**DOI:** 10.3389/fncel.2020.00226

**Published:** 2020-07-22

**Authors:** Juan Carlos Alvarado, Verónica Fuentes-Santamaría, José M. Juiz

**Affiliations:** ^1^Facultad de Medicina, Instituto de Investigación en Discapacidades, Neurológicas (IDINE), Universidad de Castilla-La Mancha, Albacete, Spain; ^2^Department of Otolaryngology, Hannover Medical School, NIFE-VIANNA, Cluster of Excellence Hearing4all-German Research Foundation, Hannover, Germany

**Keywords:** cochlear blood flow, deafness, magnesium, oxidative stress, sensorineural, vitamins

## Abstract

We live in a world continuously immersed in noise, an environmental, recreational, and occupational factor present in almost every daily human activity. Exposure to high-level noise could affect the auditory function of individuals at any age, resulting in a condition called noise-induced hearing loss (NIHL). Given that by 2018, more than 400 million people worldwide were suffering from disabling hearing loss and that about one-third involved noise over-exposure, which represents more than 100 million people, this hearing impairment represents a serious health problem. As of today, there are no therapeutic measures available to treat NIHL. Conventional preventive measures, including public awareness and education and physical barriers to noise, do not seem to suffice, as the population is still being affected by damaging noise levels. Therefore, it is necessary to develop or test pharmacological agents that may prevent and/or diminish the impact of noise on hearing. Data availability about the pathophysiological processes involved in triggering NIHL has allowed researchers to use compounds, that could act as effective therapies, by targeting specific mechanisms such as the excess generation of free radicals and blood flow restriction to the cochlea. In this review, we summarize the advantages/disadvantages of these therapeutic agents, providing a critical view of whether they could be effective in the human clinic.

## Introduction

Noise over-exposure is the major avoidable cause of permanent hearing loss (World Health Organization, 1997). As the second leading cause of adult-onset sensorineural deafness (Mathers et al., 2000), noise-induced hearing loss (NIHL) is a preventable condition that represents a large worldwide economic burden, as well as a health priority (World Health Organization, 1997, 2018; Mathers et al., 2000). It is well known that the main sources for excessive noise exposure, either alone or in combination are occupational, environmental, and recreational activities (World Health Organization, 1997, 2018; Hurtley, 2009). As far as the former is concerned, appropriate noise regulations and policies at workplaces are in effect. However, they have only been partially successful in reducing occupational-related NIHL (World Health Organization, 2018). Regarding environmental and recreational noise, the lack of awareness of the population about the hazard of over-exposure limits substantially the efficacy of regulatory measures (Śliwińska-Kowalska and Zaborowski, 2017; World Health Organization, 2018).

**Figure 1:**
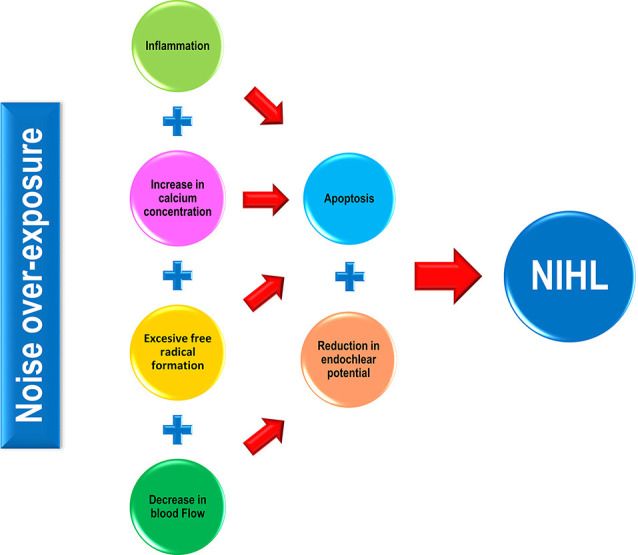
Pathophysiological mechanisms involved in noise-induced hearing loss (NIHL). In response to noise over-exposure, a cascade of inflammatory-related events occurs in the exposed cochlea, leading to hair cell apoptosis and eventually, to hearing loss. Noise-induced cochlear increases in both calcium concentration and free radical production also activate sensory cell death processes that in turn, will cause hearing impairment. Additionally, noise triggers a direct and indirect (*via* calcium) vasoconstriction in the cochlea resulting in a decline in the endocochlear potential (EP), which correlates with impaired mechanotransduction in the organ of Corti (OC) and therefore, in hearing alterations.

The importance of this issue is highlighted by the fact that the number of people exposed to noise is growing, while there is no actual medical treatment for NIHL, and conventional preventive measurements are not fully reaching their goals. There is overwhelming evidence that demonstrates that elevated calcium concentration, inflammation, increased oxidative stress, as well as a reduction in blood flow, are some of the pathophysiological mechanisms underlying NIHL (Figure 1; Lamm and Arnold, 1998; Henderson et al., 2006; Le Prell et al., 2007b; Shen et al., 2007; Bao et al., 2013; Fetoni et al., 2013; Altschuler and Dolan, 2015; Tan et al., 2016; Fuentes-Santamaría et al., 2017; Śliwińska-Kowalska and Zaborowski, 2017). It is worth noting, that at least the latter two cellular mechanisms seem to be part of a common pathogenic pathway that involves other sensorineural hearing loss conditions such as age-related hearing loss (ARHL) and drug-induced hearing loss (DIHL; Alvarado et al., 2015, 2018, 2019; Tavanai and Mohammadkhani, 2017). Therefore, therapies based on antioxidants and/or vasodilators have been postulated as strategies to prevent and/or reduce the progression of NIHL in several animal models and humans.

## Oxidative Stress and Antioxidants

**Figure 2:**
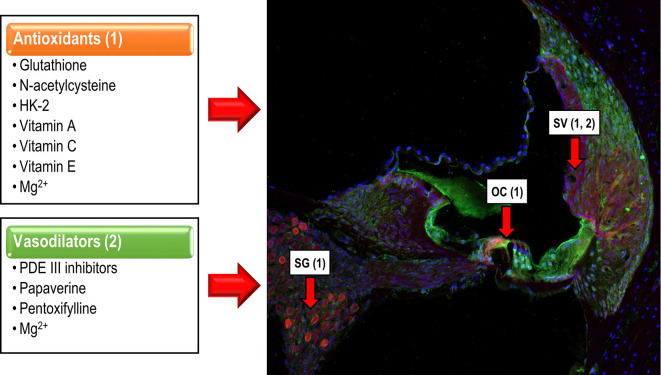
Cochlear targets of antioxidants and vasodilators. Antioxidants (1) reduce the apoptotic cochlear damage that follows noise over-exposure, targeting hair cells in the OC, neurons of the spiral ganglion (SG) and stria vascularis (SV), exerting a protective effect over the cochlea and therefore, overhearing. Vasodilators (2), act mainly on the SV, minimizing noise-induced vasoconstriction, restoring the EP, and preserving auditory function.

There is compelling evidence that suggests that noise over-exposure leads to an imbalance between excess free radical build-up, especially reactive oxygen, and nitrogen species (ROS and NRS), and its removal from cells in the auditory receptor. ROS build up in stressed mitochondria has been proposed as a major player at the origin of NIHL (Böttger and Schacht, 2013). This imbalance induces cell damage and ultimately, cell death in the organ of Corti (OC), the stria vascularis (SV) and the spiral ganglion (SG; Yamasoba et al., 1998; Henderson et al., 2006; Le Prell et al., 2007b; Fetoni et al., 2013; Altschuler and Dolan, 2015). Such reports support the hypothesis that therapeutic strategies targeting free radical overproduction are potentially useful to ameliorate the damaging effect of noise on hearing, which leads to cell death, mostly apoptotic (Figure 2). For instance, experimental studies that involve glutathione (Yamasoba et al., 1998; Ohinata et al., 2000; Hight et al., 2003; Henderson et al., 2006), N-acetylcysteine (Kopke et al., 2000, 2007), glutathione synthetic enzymes (Kil et al., 2007, 2017), multifunctional antioxidants (Chen et al., 2020) or micronutrients (Le Prell et al., 2007a,b) suggest attenuation of cochlear damage and/or reduction of the threshold shifts following noise exposure.

Consistent with this idea, glutathione, a tripeptide molecule (cysteine, glutamic acid, and glycine) and the most abundant endogenous free radical scavenger in humans, was the first antioxidant evaluated for the treatment of NIHL (Yamasoba et al., 1998; Allen and Bradley, 2011; Schmitt et al., 2015). Glutathione is involved in many metabolic processes that include scavenging of free radicals and ROS, detoxification of several products such as lipid peroxides and xenobiotics, and it also plays a crucial role in the regeneration of ascorbate and tocopherol-based antioxidants (Yamasoba et al., 1998; Ohinata et al., 2000; Hight et al., 2003; Henderson et al., 2006; Allen and Bradley, 2011; Schmitt et al., 2015). Due to its wide distribution in the cochlea, preferentially in the SV and the spiral ligament (Yamasoba et al., 1998), therapeutic strategies aimed at increasing glutathione levels have been used as a proof of principle for the treatment of NIHL in rodent models. Increased glutathione levels in noise-exposed animal models have been associated with significantly smaller threshold shifts, due to reduced cochlear damage as compared to untreated animals (Yamasoba et al., 1998; Ohinata et al., 2000; Hight et al., 2003; Henderson et al., 2006). Conversely, reduced glutathione levels increase cochlear susceptibility to noise (Yamasoba et al., 1998; Ohlemiller et al., 2000). Despite these promising results, considerations regarding glutathione availability and administration route have precluded its use as a treatment for NIHL in humans. First, while oral administration of glutathione in experimental animals seems to be useful in increasing plasma levels, such oral supplementation in humans is controversial, as γ-glutamyl transpeptidase, an intestinal enzyme, degrades exogenous glutathione before it could be absorbed (Allen and Bradley, 2011; Schmitt et al., 2015). Second, as glutathione in cells is mainly synthesized endogenously by its biochemical precursors, its parenteral administration may not result in a significant increase in the concentration of cellular glutathione levels (Hight et al., 2003).

N-acetylcysteine (NAC), a cysteine precursor, is an antioxidant molecule that enhances the production of glutathione (Kopke et al., 2000, 2007; Schmitt et al., 2015). Several studies in noise-exposed rats, chinchillas, and rabbits, have reported that the administration of NAC minimizes the progression of apoptosis in sensory hair cells and reduces significantly the auditory threshold shifts, at low and medium frequencies, as compared to untreated animals (Kopke et al., 2000; Mortazavi et al., 2010; Lu et al., 2014). In humans, the oral administration of NAC after noise exposure reduces significantly the incidence of temporary threshold shifts (TTS; Rosenhall et al., 2019) and when compared with glutathione, it has the advantage of better intestinal absorption. Thus, the available data largely support the notion that NAC could be used orally, increasing plasma levels of glutathione and acting as **a** replenisher of this endogenous scavenger (Kopke et al., 2007; Mortazavi et al., 2010; Schmitt et al., 2015). However, preliminary results suggest that high doses of NAC, still within therapeutic margins, do not limit but increase oxidative stress in the cochlea after treatment with ototoxic drugs, which share oxidative stress-related pathogenic pathways with NIHL. This finding highlights the notion that therapeutic margins for NAC, and maybe other antioxidants administered alone or in combinations, are relatively narrow and may limit synergistic interactions. It should be kept in mind that free radicals, like ROS, maybe “foes or friends.” There are physiological ROS levels that are needed for biological redox reactions to proceed in a balanced manner (Ray et al., 2012). If treatments, for instance with NAC, bring ROS levels below a threshold, redox imbalances may lead to “paradoxical” oxidative damage. Also, since glutathione synthesis is reduced with aging, the effectiveness of administered NAC decreases as subjects age (Schmitt et al., 2015). An alternative approach to potentiate glutathione-mediated mechanisms involves priming enzymatic synthesis routes. Glutathione-peroxidase1 (Gpx1) is primarily involved in glutathione synthesis in the cochlea, and reduced activity has been linked to NIHL (Ohlemiller et al., 2000; Kil et al., 2007). Ebselen, a selenium-based organic compound, mimics and potentiates Gpx1 activity, and it has been shown to reduce auditory thresholds and hair cell death when administered parenterally to rats immediately before or after NIHL (Kil et al., 2007). More recently, safety and limited but significant efficacy of Ebselen has been shown in humans (Kil et al., 2017), supporting interventions on enzymatic activities in glutathione metabolic routes as a promising therapeutic avenue.

Interestingly, recent evidence suggests that HK-2, a multifunctional antioxidant with a metal chelator and free radical scavenger properties (Kawada and Kador, 2015; Kawada et al., 2015; Chen et al., 2020), is effective in treating NIHL. Oral administration of HK-2 to Sprague-Dawley rats 10 days before noise over-exposure exerts a significant protective effect in the cochlea, not only enhancing hair cell survival but also reducing the auditory threshold shifts when compared to untreated rats. While oral intake 10 days after noise exposure did not improve overall cell survival, it still preserved auditory function (Chen et al., 2020), suggesting a mild protective effect of HK-2 when taken several days after the exposure. Given that HK-2 is efficient at reducing noise-induced cell damage, it could be administered orally and it shows no side effects, at least in rats, it has been suggested as a new treatment option for NIHL (Chen et al., 2020). However, further studies will be needed to confirm that HK-2 is a valid therapeutic alternative without adverse side effects on humans.

Several micronutrients such as vitamins A, C, and E show antioxidant properties and have been used either individually or combined as experimental treatments for NIHL. The antioxidant mechanisms of vitamins are diverse. For instance, vitamin A (an *in vivo* product derived mainly from β-carotene) is an excellent scavenger of singlet oxygen, blocking and/or reverting lipid peroxidation in the plasma membrane (Sies and Stahl, 1995; Le Prell et al., 2007a; Alvarado et al., 2015, 2018). Experimentally, when retinoic acid, the most active metabolite of vitamin A, is administered in noise-exposed mice, it protects the cochlea by reducing significantly the apoptosis of hair cells and consequently, inducing a faster recovery of the auditory thresholds when compared to untreated animals (Ahn et al., 2005, 2013; Prasad and Bondy, 2020). Vitamin E is one of the main radical scavengers of the cell membrane as it blocks and/or reverts lipid peroxidation, by reacting with, and reducing peroxyl radicals (Sies and Stahl, 1995; Le Prell et al., 2007a; Alvarado et al., 2015, 2018). Vitamin E also has a protective effect in the cochlea by reducing cell death and cellular damage in guinea pigs (Hou et al., 2003; Fetoni et al., 2008), and by diminishing the auditory threshold shifts following noise over-exposure in guinea pigs (Hou et al., 2003; Fetoni et al., 2008) and humans (Kapoor et al., 2011). Vitamin C, a water-soluble molecule, is considered a primary extracellular antioxidant and it is extremely effective in blocking and/or reverting lipid peroxidation in the plasma membrane (Sies and Stahl, 1995; Le Prell et al., 2007a; Alvarado et al., 2015, 2018). Additionally, this vitamin also protects the cell membrane by regenerating vitamin E from its oxidized form. Therefore, the oxidative functions of both vitamin C and E are coupled (Sies and Stahl, 1995; Le Prell et al., 2007a; Alvarado et al., 2015, 2018). Regarding vitamin C, its administration before noise over-exposure diminishes significantly the TTS in rats (Loukzadeh et al., 2015) and the permanent threshold shifts (PTS) in guinea pigs (McFadden et al., 2005). Interestingly, the combination of vitamins A, C, and E administered orally to patients did not show any effect over music-induced TTS (Le Prell et al., 2016). In this latter report, the authors stated that three possible explanations may account for these negative results, including participant compliance, as only one of the six doses was administered under supervision; the bioavailability of orally administered vitamins and the possible degradation of the components during the shipping process (Le Prell et al., 2016). As there are no known disadvantages associated with the oral intake of the above-mentioned antioxidant vitamins, the main advantage lays in the fact that they present good safety profiles and are free from harmful side effects (Diplock, 1995; Hathcock, 1997). Nevertheless, additional studies will be required to ascertain whether these micronutrients are beneficial for treating NIHL in humans.

## Reduction of the Cochlear Blood Flow and Vasodilators

Exposure to loud noise, in addition to inducing oxidative stress, also diminishes cochlear blood flow by reducing blood vessel diameter and erythrocyte velocity in the basilar membrane, the spiral ligament and the SV (Sendowski, 2006; Le Prell et al., 2007a, b; Alvarado et al., 2015; Shin et al., 2019). Morphologic alterations in the SV, a key structure for producing and maintaining the endocochlear potential (EP), decrease the EP and consequently, affect the cochlear amplification of acoustic signals leading to an increase in auditory thresholds, even in the absence of hair cell death (Gates and Mills, 2005; Sendowski, 2006; Schmiedt, 2010; Alvarado et al., 2015). Therefore, it is expected that therapeutic agents that improve cochlear blood flow, would also reduce auditory thresholds in NIHL, with potentially beneficial effects in patients that suffer from this condition. This is the rationale for using vasodilator drugs such as phosphodiesterase (PDE) III inhibitors, papaverine, and pentoxifylline or micronutrients like magnesium (Mg^2+^) which seem to have protective effects in the noise-exposed cochlea (Figure 2).

Accordingly, administration of milrinone, a PDE III inhibitor, to Wistar rats before noise over-exposure, ameliorates vacuolization, inflammation, and apoptotic processes in the sensory epithelium and the SV as compared to untreated rats (Ceylan et al., 2019). Although milrinone could be given orally as well as parenterally, it should be done under medical monitoring as one of its main side effects is dose-dependent cardiac arrhythmias (Davidenko and Antzelevitch, 1984; Ceylan et al., 2019). Regarding papaverine, a direct-acting vasodilator, when injected intraperitoneally in noise-exposed Wistar rats significantly decreases the apoptotic death of hair cells resulting in significantly smaller threshold shifts when compared to noise-exposed untreated animals (Kum et al., 2018). As a pharmacological agent, papaverine is not exempt from adverse effects including endothelium damage, epileptic seizures, transient neurological dysfunction, and hemodynamic changes, among others (Dipp et al., 2001; Chadwick et al., 2008). Pentoxifylline, another vasodilator compound used as a possible treatment for NIHL, has been reported to have no beneficial effects in noise-exposed guinea pigs, even though it improved the blood supply to the cochlea when administered parenterally (Lamm and Arnold, 1999). However, another study also in guinea pigs demonstrated that pentoxifylline reduced both cochlear damage and threshold shifts to values similar to untreated animals (Kansu et al., 2011; Sakat et al., 2016). Another observation to take into account is that, although pentoxifylline is a well-tolerated drug, it has several dose-related adverse effects including diarrhea, dyspepsia, constipation, confusion, seizures, hypotension, anaphylactoid reaction and hemorrhage (Tanikella et al., 2008; Hassan et al., 2014).

The micronutrient Mg^2+^ also has been proposed as a treatment for NIHL, not only for its function as a potential cochlear vasodilator but for other pharmacological properties, which include modulation of NMDA glutamate receptors and the regulation of calcium influx into hair cells, preventing or reducing apoptosis (Cevette et al., 2003; Sendowski, 2006; Le Prell et al., 2007a; Alvarado et al., 2015; Sakat et al., 2016). Further evidence supporting this idea comes from studies demonstrating that the subcutaneous injection of Mg^2+^ for one month enhanced hair cell survival and reduced significantly the auditory threshold shift in noise-exposed guinea pigs (Abaamrane et al., 2009). Similarly, the oral administration of salts of this cation, two weeks before high-level impulse-noise exposure significantly reduced the rate of acoustic trauma in guinea pigs (Scheibe et al., 2000). However, parenteral administration of Mg^2+^ alone 1 h before and up to 5 days after noise stimulation in guinea pigs, did not show reliable effects over noise over-exposure. When Mg2+ was administered combined with vitamins A, C, and E there was a substantial protective effect reflected in increased hair cell survival and decreased auditory threshold shifts (Le Prell et al., 2007a). In noise-exposed mice, a similar combination of Mg^2+^ plus vitamins A, C and E partially recovers PTS, along with a significant reduction of type II fibrocytes in the cochlear lateral wall (Le Prell et al., 2011). In humans, it has been observed that the oral administration of Mg^2+^ significantly reduced PTS (Attias et al., 1994) and TTS (Attias et al., 2004), without any notable side effect, when compared to untreated subjects. Overall, Mg^2+^ is well-tolerated although there are some dose-dependent side effects such as nausea, diarrhea, stomachache, headache, tinnitus, and dizziness, which appear when used at high concentrations (Attias et al., 1994; Sendowski, 2006; Coates, 2010).

## Conclusions

In light of new evidence about the etiopathogenic and pathophysiological mechanisms underlying NIHL, it becomes a priority to develop new therapeutic strategies that help to improve the quality of life of millions of people affected by this condition. The above-presented body of literature in this review strongly supports the fact that there are several pharmacologic agents available to prevent, at least in part, this disability. Acting preventively either on the excessive production of free radicals in the noise-exposed cochlea and/or on noise-induced cochlear vasoconstriction, they may help to ease the consequences of NIHL. It is important to keep in mind, however, that some of these substances may have pharmacological limitations, including the mechanism of drug action, administration route, tolerability, side effects, and proven efficacy, that should be considered before they could be approved for its use in this pathology. The most successful therapy, besides being efficient in reducing noise-induced cochlear damage, should be easy to administer (preferably orally), have a good safety profile, reduced drug acquisition costs, and few or no side effects. Antioxidants such as N-acetylcysteine, HK-2, vitamins A, C, and E and the vasodilator Mg^2+^ meet all these requirements acting either in different etiopathogenic pathways or on the same pathway through different targets. Therefore, it seems plausible to expect that a combination with some or all of them produces synergism and/or redundancy in their mechanisms of action, potentiating the beneficial effect over noise over-exposure. Even though these otoprotective agents represent a promising new therapeutic strategy for ameliorating, delaying, or even preventing the impact of noise on hearing, finding the ideal therapeutic agent will remain a challenging topic for the future.

## Author Contributions

JA and VF-S: drafting of the manuscript and design of figures. JA, VF-S, and JJ: critical revision of the manuscript for important intellectual content.

## Conflict of Interest

JA, VF-S, and JJ are co-inventors of the US Patents 9, 889, 156, “Method for treating NIHL” and 9, 919, 008, “Methods for treating ARHL.” Both patents are based on the use of oral ACEMg, but currently, they are not involved in any trials testing this compound or any other commercial exploitation.
